# You Are at Risk of Insomnia When You Do Not Accept Your Pre-Sleep Arousal: A Cross-Sectional Study Investigating the Relationship Between Mindfulness, Pre-Sleep Arousal and Severity of Insomnia Symptoms

**DOI:** 10.3390/brainsci15111196

**Published:** 2025-11-06

**Authors:** Marco Fabbri, Marco Mirolli, Monica Martoni

**Affiliations:** 1 Department of Psychology Renzo Canestrari, University of Bologna, 40127 Bologna, Italy; 2 Institute of Cognitive Science and Technologies, CNR, 00185 Rome, Italy; marco.mirolli@istc.cnr.it; 3 Department of Medical and Surgical Sciences (DIMEC), University of Bologna, 40126 Bologna, Italy

**Keywords:** insomnia, cognitive arousal, somatic arousal, acceptance, awareness, mindfulness

## Abstract

Background/Objectives: Psychophysiological insomnia is characterized by acquired sleep difficulties and/or a state of hyperarousal when falling asleep. This hyperarousal can develop and/or maintain insomnia. In contrast, mindfulness can reduce stress and improve sleep. This cross-sectional study aimed to assess the relationship between mindfulness traits (acceptance and awareness), pre-sleep arousal (cognitive and somatic) states, and insomnia symptoms. Methods: A sample of 464 non-clinical volunteers (352 females; mean age 27.89 ± 9.48 years) participated in this online cross-sectional study, completing the Philadelphia Mindfulness Scale (PHLMS), Pre-Sleep Arousal State (PSAS), and Insomnia Severity Index (ISI). Results: The acceptance component of mindfulness but not awareness negatively correlated with ISI, as well as PSAS subscales. In a mediation model, acceptance was associated with ISI both directly and indirectly, through associations with cognitive arousal and somatic arousal. Conclusions: This study suggests a possible mechanism by which mindfulness works to influence sleep processes. In particular, acceptance can reduce pre-sleep arousal, reducing the possibility of developing and/or maintaining insomnia symptoms.

## 1. Introduction

Insomnia is a prevalent sleep disorder characterized by persistent difficulties initiating sleep, maintaining sleep, or experiencing restorative sleep upon awakening, occurring at least three nights per week for a minimum duration of three months [1]. These symptoms must occur despite adequate opportunities for sleep and are associated with significant distress or impairment in daytime functioning [2]. While insomnia can be transient, it often becomes chronic, severely affecting quality of life and contributing to substantial psychological distress [3]. Epidemiological studies estimate that 25–30% of the general population report transient or occasional insomnia, while approximately 10–15% experience chronic insomnia [4,5,6,7]. In Italy, reported prevalence rates are notably higher, ranging from 13.4% in the earliest epidemiological studies to 64% in the large-scale “Studio Morfeo” survey [6,8,9,10,11,12,13], consistent with global reports of elevated insomnia prevalence [14,15,16,17,18,19,20]. Insomnia is frequently comorbid with other physical and mental health conditions [1,21], and is increasingly recognized as both a symptom and a potential risk factor for a wide range of medical and psychiatric disorders [22,23,24,25]. As such, targeting insomnia may serve a preventive function and positively influence the trajectory of comorbid conditions [26].

One of the most widely accepted theoretical frameworks explaining the etiology and pathophysiology of insomnia is the hyperarousal model [27,28,29,30]. According to this model, individuals with insomnia experience heightened cortical, cognitive, and physiological arousal both during the day and at night, positioning insomnia as a disorder of chronic hyperarousal [27,29]. Hyperarousal is conceptualized broadly, ranging from a stable trait-like predisposition (e.g., [31]) to a state-like elevation in arousal at sleep onset [27,28,29,30]. Physiologically, hyperarousal is associated with increased autonomic activity (e.g., elevated heart rate, altered body temperature) and dysregulated neuroendocrine functioning, such as elevated plasma cortisol and disrupted melatonin levels [32,33,34,35]. Neuroimaging studies have revealed increased global brain metabolism and altered functioning of neural circuits involved in arousal regulation, providing further support for cortical hyperarousal during sleep [36,37]. Cognitively, hyperarousal is manifested in heightened mental activity during the pre-sleep period, often marked by intrusive thoughts, rumination, and worry (e.g., “*If I don’t sleep well tonight, I won’t be able to function tomorrow*”) [38,39,40]. These maladaptive cognitive processes are further exacerbated by dysfunctional beliefs about sleep, including exaggerated concerns about the consequences of insomnia, unrealistic expectations regarding sleep needs, misconceptions about its causes, and maladaptive behaviors aimed at controlling sleep [41,42,43,44]. Several cognitive models of insomnia posit that these dysfunctional cognitions contribute to arousal, which, in turn, sustains the sleep disturbance. Notably, the cognitive model of insomnia [45] suggests that rumination and worry heighten arousal and distress, prompting selective attention to sleep-related threats. This vigilance can intensify the perceived severity of insomnia, contribute to daytime impairments, and reinforce maladaptive safety behaviors [45]. Empirical evidence supports this model, with studies showing that individuals with insomnia tend to excessively monitor their sleep environment, exhibit attentional biases toward sleep-related cues [46,47], and engage in compensatory daytime behaviors [48].

Importantly, heightened cortical, cognitive, and somatic arousal are particularly disruptive during the pre-sleep period, contributing to increased sleep latency and frequent nocturnal awakenings [49,50]. Conceptualizing arousal as a multidimensional and state-dependent construct, researchers have distinguished between cognitive and somatic components [38,39,44,51,52]. Pre-sleep hyperarousal often arises from maladaptive responses to chronic sleep difficulties, poor de-arousal capacity, and life stressors [40,45,53,54,55,56]. The Pre-Sleep Arousal Scale (PSAS) [49] assesses both cognitive (PSA-C) and somatic (PSA-S) aspects of arousal. The PSA-C reflects mental activity during bedtime, including distressing thoughts and worries, while the PSA-S captures physiological sensations that disrupt sleep. These two components are interdependent: elevated PSA-C can increase PSA-S, and vice versa [57]. Rumination may mediate this bidirectional relationship, highlighting its central role in insomnia maintenance [58].

While Cognitive Behavioral Therapy for Insomnia (CBT-I) remains the gold-standard intervention [59], Mindfulness-Based Interventions (MBIs) have gained increasing attention for their potential to alleviate insomnia, particularly in individuals with chronic health conditions [60]. Mindfulness is conceptualized as a present-focused, non-judgmental awareness of thoughts, emotions, and bodily sensations [61], which may directly target the cognitive and somatic arousal mechanisms implicated in insomnia [62]. Ong et al. [54] proposed the metacognitive model of insomnia, which emphasizes the role of metacognitive processes (i.e., secondary arousal) in perpetuating the disorder. While primary arousal involves intrusive cognitions that interfere with sleep, secondary arousal involves maladaptive responses to those cognitions, such as worry and negative appraisal [45,63]. MBIs might reduce both secondary arousal through increasing acceptance to sleep related ruminations, and primary arousal through directly decreasing stress and the problems and related cognitions dependent on it [64,65]. Though reviews support the efficacy of MBIs for improving sleep outcomes [66,67,68,69], effect sizes are modest [70,71,72,73], and the specific mechanisms underlying their effects remain under investigation.

The Monitor and Acceptance Theory (MAT) proposed by Lindsay and Creswell [74] posits that attentional monitoring enhances cognitive awareness but also affective reactivity, while the interaction between monitoring and acceptance is necessary for effective emotional regulation, the decrease affective reactivity, and the improvement of stress-related health outcomes. Evidence suggests that while awareness alone may increase psychological distress, its negative impact can be mitigated by acceptance [75,76]. However, a thorough analysis of the relevant literature shows that the evidence for the MAT theory is highly inconsistent, and an alternative theory, according to which the positive effects of mindfulness depend almost exclusively on acceptance, is much more supported by available evidence [77]. Regarding sleep, in particular, a series of recent studies have consistently shown that it is indeed acceptance, and not attention monitoring, that is responsible for reducing psychological distress and improving sleep [77,78,79]. These results align with the metacognitive model of insomnia [54], implying that acceptance reduces secondary arousal, thereby attenuating primary arousal and facilitating sleep.

**Figure 1:**
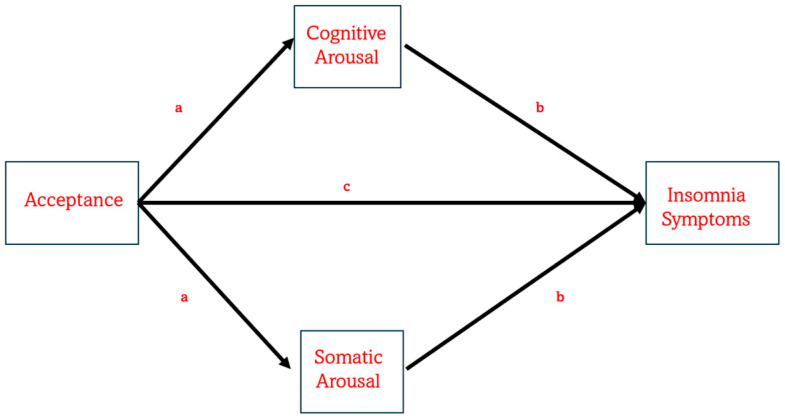
The figure illustrates the expected model explaining how mindfulness acceptance can be directly associated with the severity of insomnia symptoms. Also, the model hypothesizes that acceptance is associated with insomnia symptoms indirectly via cognitive and/or somatic arousal. In this model, several covariates are taken into account, such as sociodemographic information and meditation practices.

Notably, previous studies relied on subscales from the Five Facet Mindfulness Questionnaire (FFMQ) [80], which do not distinctly capture the constructs of attention monitoring and acceptance. Specifically, the former component is represented by Observing subscale score, while the latter by two Non-judgment and Non-reacting subscales are associated with acceptance [77,78,79]. In contrast, the Philadelphia Mindfulness Scale (PHLMS) [81] includes only two separate subscales, measuring awareness, which is equated to attention monitoring, and acceptance, thus offering a more precise measure for exploring their independent contributions. To the best of our knowledge, no previous studies tested the association between acceptance or awareness and insomnia, using PHLMS. In addition, and more importantly, no prior studies have yet tried to address how acceptance and awareness are connected to hyperarousal in predicting insomnia. The present study aimed to fill this gap. Specifically, this cross-sectional study aimed to examine, in a non-clinical population, whether the acceptance and awareness components of mindfulness predict insomnia severity, and whether this relationship is mediated by cognitive and somatic pre-sleep arousal (PSA-C and PSA-S), as shown in Figure 1. On the basis of the studies just discussed, we hypothesized that acceptance would predict insomnia severity (measured by the Insomnia Severity Index; ISI [82]) indirectly through PSA-C and/or PSA-S (Figure 1). In contrast, we expected that awareness would not significantly predict ISI scores, either directly or indirectly via the proposed mediators.

## 2. Materials and Methods

### 2.1. Participants

A total of 464 volunteers participated in the online study conducted via PsyToolkit [83,84]. A web-based approach was employed to maximize sample size and minimize the risk of underestimating statistical power [85]. Participation was anonymous and unpaid, and individuals could withdraw at any time without penalty.

Of the total sample, 352 participants identified as women, 109 as men, and 3 participants did not disclose their gender. The mean age of the sample was 27.89 years (SD = 9.48 years). Regarding education, 47.60% of participants held a bachelor’s degree, 28.70% held a high school diploma, 15.50% held a master’s degree, 5.80% reported postgraduate qualifications (e.g., university master’s or PhD). Additionally, 2.40% had completed only middle school. The gender differences for age and education level are reported in Table 1.

Current engagement in meditation (present meditation) was reported by 8.60% of participants, with a mean practice duration of 20.20 months (SD = 32.52), a weekly average of 78.90 min (SD = 108.36), and a mean session length of 19.45 min (SD = 20.87). A higher percentage (22.80%) reported previous meditation experience (past meditation), with an average duration of 5.32 months (SD = 7.86), a weekly mean of 51.08 min (SD = 46.57), and an average session length of 21.21 min (SD = 16.28). As expected, age positively correlated with the duration of past meditation practice (*r* = 0.22, *p* = 0.025), but no significant association emerged between age and current meditation duration (*r* = 0.25, *p* = 0.12). In Table 1, the differences between males and females for present and past meditations practices are presented.

The study protocol was approved by the Ethics Committee of the Department of Psychology at the University of Campania Luigi Vanvitelli, where the corresponding author was affiliated at the time of the study. All participants provided informed consent through four distinct consent forms prior to participation.

### 2.2. Insomnia Severity Index (ISI)

Participants completed the Insomnia Severity Index (ISI), which assesses insomnia symptoms over the past two weeks [86]. The Italian version of the ISI [87], consisting of 7 items rated on a 5-point Likert scale (0–4), was used. Total scores range from 0 to 28, with higher scores reflecting more severe insomnia. Based on validated cut-off criteria [82,86,87], 52.60% of the sample exhibited no clinically relevant insomnia (0–7 range), 33.60% had subthreshold insomnia (8–14 range), 12.70% moderate insomnia (15–21 range), and 1.10% severe insomnia (22–28 range). The internal consistency was good (Cronbach’s α = 0.85).

### 2.3. Pre-Sleep Arousal Scale (PSAS)

The Italian version of the Pre-Sleep Arousal Scale (PSAS) [88] was used to assess pre-sleep arousal over the previous two weeks [49]. The PSAS consists of 16 items, divided into two subscales: Somatic Arousal (PSA-S; 8 items, e.g., “*heart racing, pounding, or beating irregularly*”) and Cognitive Arousal (PSA-C; 8 items, e.g., “*being mentally alert, active*”). Items are rated on a 5-point Likert scale (1 = not at all to 5 = extremely), with total scores ranging from 8 to 40 per subscale. Higher scores for both subscales indicate higher arousal at the moment of going to bed. Internal consistency was confirmed for both subscales (PSA-S α = 0.82; PSA-C α = 0.84).

### 2.4. Philadelphia Mindfulness Scale (PHLMS)

Participants also completed the Italian version of the Philadelphia Mindfulness Scale (PHLMS) [81,89]. This 20-item self-report measure assesses two core components of mindfulness: present-moment awareness (10 items, e.g., *“When talking with other people, I am aware of their facial and body expressions*”) and acceptance (10 items; all reversed, e.g., “*I try to stay busy to avoid thoughts and feelings from coming to mind*”). Responses are given on a 5-point Likert scale (1 = never to 5 = very often), with each subscale ranging from 10 to 50. Higher scores for both subscales indicate higher mindfulness component. The PHLMS demonstrated good internal consistency in the current sample (overall α = 0.81; awareness α = 0.79; acceptance α = 0.89).

### 2.5. Procedure

The study was disseminated through psychology courses of bachelor’s or master’s degree and major social media channels. Interested participants received the survey link. When the link was sent to interested participants, he/she was invited to forward the link to parents and/or friends with a brief (written) presentation of the study, in line with a snowball sampling technique. The PsyToolkit platform enforced participation via desktop or laptop only, excluding mobile devices to ensure stimulus consistency. No specific exclusion criteria were applied, apart from the requirement that participants be proficient in Italian and of legal age, as both were necessary for completing the self-report measures and providing informed consent.

The survey sequence began with a study description and instructions. Informed consent was then obtained through a series of four separate agreements. Participants first provided demographic and meditation-related information, followed by completion of the ISI, PSAS, and PHLMS. A debriefing text and contact information for further inquiries were provided at the end.

### 2.6. Data Analysis

Although the validation studies of questionnaires used in the present study provided evidence for the assumption of normality of questionnaire scores [49,81,82,86,87,88,89], we decided to transform all scores using the natural logarithmic (ln) procedure, in order to solve any problems relating to normal distribution or the type of distribution for skewness and kurtosis. However, in the result section, we reported raw scores or data. Based on ISI cut-off scores, a between-subjects MANCOVA was conducted to compare PSAS (and relative subscales) and PHLMS (and relative subscales) across insomnia severity groups, using gender, age, education level, and meditation experience as covariates. Partial correlations (controlling for sociodemographic variables and meditation experience) were then computed across variables. Finally, mediation analyses were conducted using Hayes’ PROCESS macro [90], Model 4. Unstandardized indirect effects were estimated using 5000 bootstrap samples, and 95% bias-corrected confidence intervals excluding zero were interpreted as evidence of mediation. The model tested whether awareness and/or acceptance were associated with PSAS, which, in turn, were hypothesized to predict ISI scores, controlling for gender, age, educational level and meditation experience.

## 3. Results

First of all, we decided to assess the presence of gender differences in sociodemographic information and meditation experience (Table 1). In addition, we performed a Spearman correlation analysis to test the associations between age, educational level, duration in months, weekly duration in minutes, and mean session duration in minutes of both present and past meditation experiences (Table 2). A gender difference was found for age, educational level, duration of past meditation experience, ISI, PSAS-S, total PSAS, Acceptance and Awareness (Table 1). Moreover, we found that age and educational level were positively associated with acceptance and PHLMS scores. Finally, only duration (in months) of present meditation practice was positively associated with the awareness component of mindfulness (Table 2).

Given that only five participants in our sample reported severe insomnia symptoms, these individuals were included in the moderate insomnia group to ensure sufficient statistical power. This inclusion was also possible considering that both moderate and severe insomnia groups reflected clinically relevant insomnia symptoms [82,86]. The MANCOVA revealed a difference among ISI group for PHLMS acceptance and PHLMS score, and all PSAS scores, as presented in Table 3. Post hoc analyses indicated that participants with no clinically significant insomnia reported significantly higher acceptance and PHLMS total scores than both the subthreshold and moderate/severe insomnia groups (*p* < 0.0001), and the subthreshold group scored higher on acceptance than the moderate/severe group (*p* < 0.001). As shown in Table 3, individuals in the moderate/severe insomnia group exhibited significantly higher total PSAS, PSA-C and PSA-S scores than those in the other two ISI groups (*p* < 0.001 for all comparisons), while participants in the subthreshold group scored higher than those in the no insomnia group on all PSAS scores (*p* < 0.0001 for all comparisons).

These findings were further supported and extended by the partial correlation analyses (see Table 4). In addition to the expected positive correlations between ISI scores and both PSAS subscales, results indicated that the acceptance component and total PHLMS score was negatively associated with ISI, PSA-C, and PSA-S scores. Consistent with the MANCOVA results, the awareness component of mindfulness was not significantly associated with any of the other variables (Table 4).

**Figure 2:**
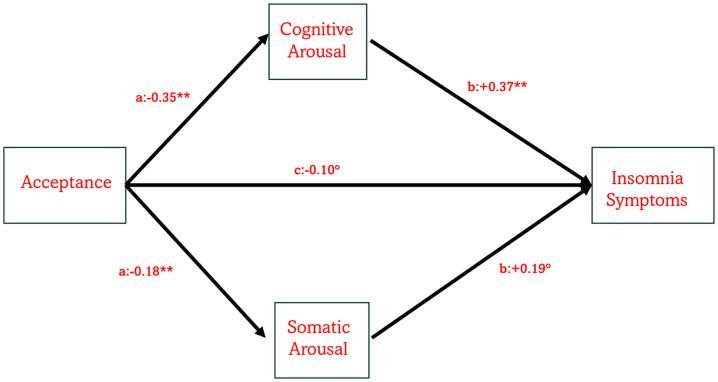
The model illustrates the role of mindfulness acceptance in predicting the severity of insomnia symptoms both directly and indirectly via PSA-C and PSA-S. Note: ° indicates *p* = 0.005, while ** indicates *p* = 0.0001.

Considering that in both MANCOVA and partial correlations awareness was not associated with ISI and PSAS, we decided to test only how acceptance component of mindfulness was directly and indirectly associated with ISI through PSAS. Figure 2 presents the results of the mediation model. When mindfulness acceptance was entered as the predictor (Figure 2), the model was statistically significant (*R*^2^ = 0.14, *F*(6, 432) = 12.14, *p* < 0.0001). A significant direct effect of acceptance on ISI scores was observed (*β* = −0.10, *t* = −3.12, *p* = 0.0019), with a 95% confidence interval (CI) ranging from −0.15 to −0.04. Additionally, two significant indirect effects emerged: a) Acceptance → PSA-C → ISI, with *β* = −0.13 (95% CI [−0.17, −0.09]); b) Acceptance → PSA-S → ISI, with *β* = −0.03 (95% CI [−0.06, −0.01]). The indirect model was significant with *R*^2^ = 0.39, *F*(8, 430) = 34.22, *p* < 0.0001.

## 4. Discussion

The aim of the present study was to investigate whether, and how, the two components of mindfulness of acceptance and awareness were associated with the severity of insomnia symptoms, either directly or indirectly, through pre-sleep arousal. Specifically, we hypothesized that the acceptance component would be associated with ISI scores both directly and indirectly via other variables, whereas awareness would not significantly contribute to the model [54,64,65,66,67,68,70,77,78,79]. To this end, a large non-clinical sample participated in an online cross-sectional study. Participants completed the Insomnia Severity Index (ISI) [82,87]; the Pre-Sleep Arousal Scale (PSAS), which measures both cognitive and somatic arousal [49,88]; and the Philadelphia Mindfulness Scale (PHLMS), assessing mindfulness components of *acceptance* and *awareness* [80,89].

Using both categorical and continuous analytical approaches, we consistently found that acceptance, but not awareness, was associated with insomnia symptoms. Specifically, when participants were grouped based on ISI scores, the moderate/severe insomnia group reported lower acceptance (and total PHLMS) scores, followed by the subthreshold insomnia group, with the no clinical group reporting the highest acceptance scores. This negative association was further supported by correlation analyses [54,62,64,65,66,67,68,69,70,71,73,77,78,79]. These findings highlight the importance of accepting, rather than avoiding or controlling, negatively perceived thoughts, emotions, and physical sensations. Acceptance involves non-judgmentally observing one’s internal experiences and viewing them as transient events [91]. This aligns with the metacognitive model of insomnia [54], suggesting that cultivating an accepting attitude may reduce avoidance of sleep-related stimuli, worry, and anxiety. Consistent with this, the moderate/severe insomnia group reported significantly higher levels of PSAS score as well as both cognitive (PSA-C) and somatic (PSA-S) pre-sleep arousal, indicating heightened mental activity and physiological tension before sleep [21,27,28,29,30,31,33,41,44,45,46,47,48,49,50,51]. Correlation analyses confirmed these results, showing a positive association between ISI scores and both PSA-C and PSA-S scores [49,50,51], lending further support to the hyperarousal model of insomnia [21,27,28,29,30,31,32,33,34,35,36,37,38,39,40,41,42,43,44,45,48].

The most compelling results emerged from the mediation model. As shown (and expected) in Figure 2, acceptance was associated with insomnia symptoms both directly and indirectly via pre-sleep arousal. Two indirect pathways were identified: (a) acceptance was negatively associated with PSA-C, which in turn was positively associated with ISI scores; (b) acceptance negatively predicted PSA-S, which in turn was positively associated with ISI scores. The first pathway underscores the importance of cognitive arousal in the persistence of insomnia. Cognitive processes such as maladaptive beliefs about sleep, repetitive thinking, worry, and rumination are known to exacerbate sleep difficulties [38,40,41,42,43,44,45,46,47,48,54,55,56,57,62,63,64,65,66,67,68,69,70,71,72,77,78,79]. Learning to accept such thoughts non-judgmentally may reduce their impact by fostering an attitude of openness and compassion toward one’s experiences. As conceptualized by mindfulness theory, individuals high in acceptance “*experience events fully and without defence, as they are*” [92], remaining present without clinging to belief or disbelief [81]. The second pathway seems to confirm the hyperarousal model [27,28,29,30] given that low level of acceptance determined an increase in somatic arousal, such as elevated heart rate, muscle tension, or breathing difficulties, which can further hinder sleep onset and maintenance, thereby aggravating insomnia [49]. Acceptance may interrupt this process by promoting a non-reactive stance toward internal experiences and reducing anxiety and avoidance responses (e.g., [93]). This pathway is in line with the general assumption that mindfulness improves the quality of sleep by reducing stress [65] and further suggests that acceptance is a crucial mindfulness aspect to act on physiological alterations reported in insomnia patients [21,24,25,31,32,33,34]. Future research should replicate these findings using alternative paradigms, such as clinical trials or observational studies.

In contrast, as expected, our analyses revealed no significant role for the awareness component of mindfulness in predicting ISI scores, PSA-C and PSA-S. This result confirmed previous studies, demonstrating that awareness has no (or at least little) role for sleep disorders [77,78,79]. Although prior studies have consistently reported no effect of awareness on insomnia, sleep problems, or sleep quality [77,78,79], we considered it relevant to further examine this association. Indeed, in the present study, awareness was assessed using the PHLMS, which confirmed the findings previously obtained with the FFMQ Observing subscale [77,78,79].

Limitations of this study must be acknowledged. First, our sample was not representative of the general population, as it was largely composed of university students, and social media users, due to the online procedure adopted, with imbalances in gender (i.e., more females), age (i.e., more young adults), and education (i.e., more educated participants). Although we controlled for these socio-demographic variables in statistical analyses, future studies should recruit more diverse and balanced samples. Second, due to the cross-sectional design, causal relationships cannot be established; longitudinal research or experimental study is warranted. Finally, sleep quantity and quality were not objectively assessed, nor were primary and secondary arousal explicitly measured. Future studies should include more precise measures of arousal as conceptualized in the metacognitive model.

## 5. Conclusions

In conclusion, we conducted a study combining self-report questionnaires assessing pre-sleep arousal (PSAS), insomnia symptoms (ISI), and two components of mindfulness (PHLMS). In an effort to elucidate a potential mechanism through which mindfulness may influence insomnia severity, our findings consistently demonstrated that acceptance is associated with insomnia symptoms. This association has been repeatedly found using different statistical analyses and procedures. Importantly, the mediation model demonstrated that acceptance predicted ISI scores both directly and indirectly via pre-sleep arousal components independently. These findings not only reinforce the reliability of the hyperarousal framework in explaining the development and maintenance of insomnia symptoms [21,27,28,29,30,31,32,33,34,35,36,37,38] but also highlight the central role of cognitive processes in the severity of insomnia [39,40,41,42,43,44,45,46,47,48]. Moreover, the model provides preliminary support for the metacognitive model of insomnia [54,55,57], offering a plausible mechanism by which mindfulness, and specifically the acceptance component, may modulate sleep disturbances. Finally, with respect to mindfulness theory, our results confirm previous research showing that the beneficial effects of mindfulness depend on acceptance [77] with awareness playing no role, as previously hypothesized [74]. Interestingly, our study suggests that acceptance may interrupt the vicious cycle of pre-sleep arousal by facilitating both cognitive and physiological deactivation. This finding points to a potential mechanism underlying the treatment of insomnia.

From a clinical perspective, these results indicate that mindfulness-based interventions (MBIs) may serve as a valuable adjunct to cognitive behavioural therapy for insomnia (CBT-I) [94]. Such interventions can help individuals cultivate a different relationship with their present-moment experiences, fostering acceptance rather than avoidance of distressing thoughts, emotions, and bodily sensations. The combined application of MBIs and CBT-I could be particularly beneficial for patients whose sleep-interfering processes involve an excessive reliance on controlled information processing. Alternatively, a synergistic approach could be adopted to promote cognitive deactivation and greater acceptance of spontaneous physiological and mental processes. Thus, acceptance-based MBIs may facilitate a more adaptive attitude toward spontaneously occurring physical and cognitive events, ultimately promoting sleep. The findings of the present study could also inform clinical practice among healthcare professionals (e.g., general practitioners and sleep clinicians), potentially reducing reliance on pharmacological treatments, enhancing treatment adherence, and improving overall clinical responsibility. Moreover, the practical implications of this study extend to the development of new mindfulness-based mobile applications. Indeed, there is increasing evidence of the growing popularity of mobile apps and online programs [95] that provide guided meditations, sleep stories, and breathing exercises designed to reduce stress, improve focus, and enhance mental well-being. Given that most of these programs are primarily based on mindfulness-based stress reduction (MBSR), the present findings suggest that acceptance-focused MBIs could further improve sleep quality—both directly, by reducing pre-sleep arousal, and indirectly, by fostering a more accepting mindset.

Future research should aim to more precisely examine the metacognitive model of insomnia and clarify the mechanisms through which mindfulness exerts its therapeutic effects on sleep disturbances. A deeper understanding of these mechanisms could have a significant societal impact, considering the global prevalence of insomnia and its substantial social, economic, occupational, and health-related consequences.

## Figures and Tables

**Table 1:** The descriptive statistics for socio-demographic and questionnaires for males and females, as well as for general sample, are presented. The statistical comparisons between males and females are also reported. The statistical results are shown in bold.

		Gender			
		M	F	Total	Comparison
**Age** **(years)**		29.61 (10.98)	27.29 (8.88)	27.89 (9.48)	***t*** **(459) = 2.25, ** ***p*** ** = 0.025,** ***Cohen’s d*** ** = 0.23**
**Education Level (%)**	No education	0%	0%	0%	***χ*** ***^2^*** **(5) = 15.35, ** ***p*** ** = 0.009,** ***w*** ** = 0.18**
	Middle School	3.70%	1.70%	2.40%	
	High School	34.90%	26.70%	28.70%	
	Bachelor’s Degree	37.60%	50.90%	47.60%	
	Master’s Degree	15.60%	15.60%	15.50%	
	PhD or Equivalent	8.20%	5.10%	5.80%	
**Present** **Meditation**	% of Practice	8.30%	8.50%	8.60%	*χ^2^*(2) = 2.35, *p* = 0.31, *w* = 0.07
	Duration in Months	23.67 (38.85)	19.43 (31.61)	20.20 (32.52)	*t*(37) = 0.33, *p* = 0.74, *Cohen’s* *d* = 0.12
	Weekly Duration in Minutes	98.89 (107.52)	72.87 (111.54)	78.90 (108.36)	*t*(37) = 0.62, *p* = 0.54, *Cohen’s d* = 0.24
	Mean Session Length in Minutes	20.89 (17.35)	19.03 (22.41)	19.45 (20.87)	*t*(37) = 0.23, *p* = 0.82, *Cohen’s* *d* = 0.09
**Past Meditation**	% of Practice	22.30%	23.10%	22.80%	*χ^2^*(2) = 0.62, *p* = 0.74 *w* = 0.04
	Duration in Months	8.20 (10.08)	4.46 (6.92)	5.32 (7.86)	***t*** **(98) = 2.03, ** ***p*** ** = 0.045,** ***Cohen’s d*** ** = 0.43**
	Weekly Duration in Minutes	57.48 (55.19)	49.17 (43.90)	51.08 (46.57)	*t*(98) = 0.75, *p* = 0.46, *Cohen’s d* = 0.17
	Mean Session Length in Minutes	22.70 (18.10)	20.77 (15.80)	21.21 (16.28)	*t*(37) = 0.50, *p* = 0.62, *Cohen’s* *d* = 0.11
**ISI**		8.78 (5.84)	7.70 (5.25)	7.93 (5.40)	***t*** **(459) = 3.81, ** ***p*** ** = 0.0001,** ***Cohen’s d*** ** = 0.19**
**PSAS-C**		19.46 (5.77)	20.03 (6.28)	12.76 (4.97)	*t*(459) = −0.84, *p* = 0.40, *Cohen’s* *d* = 0.09
**PSAS-S**		11.50 (4.01)	13.16 (5.18)	19.88 (6.17)	***t*** **(459) = −3.09, ** ***p*** ** = 0.002,** ***Cohen’s d*** ** = 0.36**
**Total PSAS**		30.95 (8.66)	33.19 (10.14)	32.64 (9.58)	***t*** **(459) = −2.08, ** ***p*** ** = 0.038,** ***Cohen’s d*** ** = 0.24**
**Acceptance**		29.79 (7.35)	26.71 (7.36)	27.47 (7.53)	***t*** **(459) = 3.81, ** ***p*** ** = 0.0001,** ***Cohen’s d*** ** = 0.42**
**Awareness**		35.28 (5.09)	37.28 (5.44)	36.76 (5.50)	***t*** **(459) = −3.41, ** ***p*** ** = 0.001,** ***Cohen’s d*** ** = 0.38**
**Total PHLMS**		65.06 (9.09)	63.99 (9.54)	64.23 (9.41)	*t*(459) = 1.04, *p* = 0.30, *Cohen’s d* = 0.11

**Table 2:** Spearman’s *r* correlation coefficients are reported of the associations between sociodemographic information as well as meditation experience (duration of practice in month, weekly duration in minutes and mean session duration in minutes) with insomnia severity index (ISI), pre-sleep arousal scale (PSAS-C for cognitive arousal, PSAS-S for somatic arousal, and Total PSAS for total score), Philadelphia Mindfulness Score (acceptance and awareness components as well as Total PHLMS score). Statistically significant correlations are indicated in bold.

	ISI	PSAS-C	PSAS-S	Total PSAS	Acceptance	Awareness	Total PHLMS
**Age**	+0.05	−0.09	−0.05	−0.08	+0.13 *	−0.005	**+0.09 °**
**Educational Level**	−0.05	−0.04	−0.07	−0.06	+0.16 *	+0.05	+0.15 *
**Duration in Month of** **Present Meditation**	+0.16	+0.09	−0.06	+0.001	−0.13	**+0.37 °**	+0.08
**Weekly Duration in** **Minutes of Present** **Meditation**	+0.18	+0.01	+0.003	+0.02	+0.07	+0.27	+0.18
**Mean Session Duration in** **Minutes of Present** **Meditation**	+0.09	−0.11	+0.07	−0.01	+0.25	−0.05	+0.16
**Duration in Month of** **Past Meditation**	−0.03	−0.05	+0.04	−0.03	+0.10	+0.05	+0.15
**Weekly Duration in** **Minutes of Past Meditation**	−0.13	−0.11	−0.16	−0.14	+0.01	+0.08	+0.07
**Mean Session Duration in** **Minutes of Past Meditation**	−0.022	+0.08	+0.11	+0.11	−0.06	+0.16	+0.06

Note: ° *p* < 0.05 and * *p* < 0.01.

**Table 3:** Means and standard deviations (SDs) for the PHLMS, PSAS, and relative subscales in each ISI group. Additionally, MANCOVA results (*F*-values, *p*-values, and partial eta-squared or *η^2^_p_* effect sizes) are presented. Statistically significant results are highlighted in bold.

Variables	No Clinical Insomnia	Subthreshold Insomnia	Moderate/Severe Insomnia	*F*(2,451)=	*p* Value	*η^2^_p_* Value
**Acceptance**	29.37 (7.10)	26.54 (7.30)	22.97 (6.79)	**23.54**	**0.0001**	**0.10**
**Awareness**	37.14 (5.71)	36.30 (5.24)	35.71 (5.81)	1.26	0.28	0.006
**PHLMS**	66.51 (8.82)	62.84 (9.49)	58.68 (8.21)	**21.02**	**0.0001**	**0.09**
**PSA-C**	17.17 (5.37)	21.67 (5.22)	26.05 (5.43)	**80.35**	**0.0001**	**0.27**
**PSA-S**	10.99 (3.76)	14.13 (5.30)	15.85 (5.04)	**47.04**	**0.0001**	**0.18**
**PSAS**	28.16 (7.95)	35.80 (9.12)	41.90 (8.23)	**87.97**	**0.0001**	**0.29**

**Table 4:** Pearson’s *r* correlation coefficients are reported, controlling for gender, age, educational level, and meditation experience. Statistically significant correlations are indicated in bold.

	ISI	Acceptance	Awareness	PHLMS	PSA-C	PSA-S	PSAS
**ISI**	**1**	−0.34 *	−0.05	−0.31 *	+0.59 *	+0.48 *	+0.61 *
**Acceptance**	-	**1**	+0.006	+0.79 *	−0.42 *	−0.29 *	−0.41 *
**Awareness**	-	-	**1**	+0.60 *	−0.004	+0.007	−0.003
**PHLMS**	-	-	-	**1**	−0.35 *	−0.24 *	−0.34 *
**PSA-C**	-	-	-	-	**1**	+0.58 *	+0.93 *
**PSA-S**	-	-	-	-	-	**1**	+0.84 *
**PSAS**	-	-	-	-	-	-	**1**

Note that * *p* < 0.0001.

## Data Availability

The data presented in this study are available on request from the corresponding author due to privacy concerns.
